# Review of Assessment Scales for Diagnosing and Monitoring Sports-related Concussion

**DOI:** 10.7759/cureus.1922

**Published:** 2017-12-07

**Authors:** Alexa M Dessy, Frank J Yuk, Akbar Y Maniya, Alex Gometz, Jonathan J Rasouli, Mark R Lovell, Tanvir F Choudhri

**Affiliations:** 1 Icahn School of Medicine at Mount Sinai, Mount Sinai Medical Center; 2 Concussion Management of New York, Icahn School of Medicine at Mount Sinai; 3 ImPACT Applications, Inc., UPMC

**Keywords:** concussion, sports-related injury, impact, sac, scat3

## Abstract

Sports-related concussion has emerged as a public health crisis due to increased diagnosis of the condition and increased participation in organized and recreational athletics worldwide. Under-recognition of concussions can lead to premature clearance for athletic participation, leaving athletes vulnerable to repeat injury and subsequent short- and long-term complications. There is overwhelming evidence that assessment and management of sports-related concussions should involve a multifaceted approach. A number of assessment criteria have been developed for this purpose. It is important to understand the available and emerging diagnostic testing modalities for sports-related concussions. The most commonly used tools for evaluating individuals with concussion are the Post-Concussion Symptom Scale (PCSS), Standard Assessment of Concussion (SAC), Standard Concussion Assessment Tool (SCAT3), and the most recognized computerized neurocognitive test, the Immediate Post-Concussion Assessment and Cognitive Testing (ImPACT). The strengths and limitations of each of these tools, and the Concussion Resolution Index (CRI), CogSport, and King-Devick tests were evaluated. Based on the data, it appears that the most sensitive and specific of these is the ImPACT test. Additionally, the King-Devick test is an effective adjunct due to its ability to test eye movements and brainstem function.

## Introduction and background

An estimated 38 million children and adolescents participate in organized sports in the United States (US), and approximately 170 million adults participate in some form of athletic activity [1]. Up to 3.8 million traumatic brain injuries (TBI) occur in this country each year, and over 300,000 of these injuries occur due to sports and recreational activities [1]. These values are likely underestimated, as 50% of concussions may go unreported [2]. With approximately 5.3 million US residents living with TBI-related disabilities, including long-term cognitive and psychological deficits, the importance of effective prevention and management strategies is clear [3].

A concussion is a transient disturbance of brain function caused by head trauma, which involves complex neurometabolic processes [4]. Evidence suggests that the concussed brain is less responsive to normal neural activation and that engagement in cognitive and physical activities prior to complete recovery can cause prolonged brain dysfunction. Under-recognition of concussions can lead to premature clearance for athletic participation, leaving athletes vulnerable to repeat injuries. Catastrophic and long-term consequences of concussions, such as second impact syndrome (SIS) and chronic traumatic encephalopathy (CTE), though rare, have been observed to occur as a result of premature return-to-play and highlight the need for a greater understanding of the mechanisms of concussions and improvement of prevention strategies [5].

Different assessment criteria have been developed to assist in the early recognition of sports-related concussions. The most commonly used assessments for evaluating individuals with concussions are the Post-Concussion Symptom Scale (PCSS), Standard Assessment of Concussion (SAC), Standard Concussion Assessment Tool (SCAT3), and the Immediate Post-Concussion Assessment and Cognitive Testing (ImPACT) [6]. This article will discuss the strengths and limitations of each of these tools, as well as the Concussion Resolution Index (CRI), CogSport, and King-Devick (KD) tests.

Symptom severity, neuropsychological function, and postural stability do not appear to be related or affected to the same degree after concussion [7]; therefore, the assessment and management should be multi-faceted. The evaluation includes a clinical exam, self-reported symptom checklist, postural assessment, and neurocognitive testing [8-10]. In particular, evaluation of cognitive functioning should include intellectual functioning, academic skills, attention and concentration, processing speed and learning, memory, psychomotor function, and emotional functioning [11]. To facilitate a comprehensive assessment of concussed athletes, several assessment batteries, such as SCAT3 and ImPACT, can be easily and rapidly administered over multiple testing sessions.

## Review

Methodology

Multiple literature searches were conducted with search criterion being an assessment of sports-related concussion. Subsequent searches were performed with the search criterion being the names of the different tests being analyzed and their efficacy (PCSS, SAC, SCAT3, ImPACT, CRI, CogSport, and KD tests). Twenty-four articles were identified, and their categorical and statistical data were analyzed.

Post-Concussion Symptom Scale (PCSS)

While new, sophisticated technologies and testing methods have been developed, symptom checklists and scales remain the standard instruments used by clinicians to evaluate concussions. They are employed as an objective tool to assess the various concussion-related symptoms and measure their severity over serial evaluations [7, 12].

One of the most commonly used symptom evaluations is the PCSS, which received endorsement by the International Symposium for Concussion in Sport, and the Graded Symptom Checklist (GSC), which is recommended by the National Athletic Trainer’s Association [7, 13]. The PCSS includes a battery of concussion-related symptoms (including headache, nausea, vomiting) and a severity scale from 0 - 6 with 0 being none and 6 being severe [14]. It has a reported sensitivity of 40.81%, specificity of 79.31%, a positive predictive value of 62.50%, and a negative predictive value of 61.33% [15]. The limitations of PCSS include the intrinsic subjective nature of a self-reported questionnaire. In addition, some evidence suggests a wide range of variability on PCSS shown among concussed individuals [12]. Due to these limitations, PCSS and other symptoms scales should not be used in isolation.

The Standardized Assessment of Concussion (SAC)

The SAC is a five to 10-minute paper and pencil test. It is a neuropsychological assessment tool developed to identify the effects of mild traumatic brain injury on the sideline and does not require specific training in neuropsychology for the purposes of administration or interpretation. The test assesses orientation, immediate recall, concentration, and delayed recall [16]. Performance on each component is summed for a total possible score of 30. Performance decrements of one point or more are consistent with impaired cognitive functioning following concussion. Previous studies have supported the validity, accuracy, and reliability of this tool as a test for determining the presence of a concussion [6, 16]. In particular, the SAC has been shown to have a sensitivity of 80 - 94% and a specificity of 76 - 91% [17-18]. This test can be repeated over time to track recovery and is used to supplement other diagnostic assessments.

This tool alone is insufficient to make return-to-play decisions. Less emphasis should be placed on the numerical SAC score and more on the use of each SAC component to evaluate neurocognition [6]. The SAC test reliably identified mild TBI symptoms for all children aged six years and older who presented to the pediatric emergency department [2]. Most college athletes will return to baseline performance on the SAC within 48 hours of injury [15].

The Standardized Concussion Assessment Tool (SCAT3)

The SCAT3 combines aspects of several concussion tools, including the PCSS, into eight components designed to assess concussion symptoms, cognition, and neurological signs. Each of the eight components is scored and recorded [9]. The newest version, the SCAT3, is a product of the 2012 Zurich Conference and serves as a standardized tool for evaluating injured athletes for concussions on the sidelines. It can be used in athletes aged 13 years and older [19]. The test consists of the Glasgow Coma Scale (GCS), Maddocks score, symptom evaluation, cognitive evaluation using SAC, neck examination, balance examination, coordination examination, and a follow-up of the SAC delayed recall task. The SCAT3 is not meant to replace comprehensive neuropsychological testing. It should not be used as a stand-alone method to diagnose concussion, measure recovery, or make decisions about an athlete’s readiness to return to competition after a concussion [20].

The Child SCAT3 was developed because children need different tools for symptom assessment and mental status testing; their balance and coordination are also different than older athletes [21]. The Child SCAT3 also includes the Glasgow coma scale, the Child Maddocks score, a child report, a parent report, cognitive assessment using the SAC, neck examination, balance examination using the Balance Error Scoring System (BESS) and tandem gait, coordination examination of the upper limbs, and the delayed recall portion of the SAC.

Immediate Post-Concussion Assessment and Cognitive Testing (ImPACT)

ImPACT is a 20-25 minute computer-based assessment tool comprised of six modules, which produce four output scores, including verbal memory, reaction time, visual-motor speed, and visual-memory composites [22]. ImPACT collects demographic data, performs neuropsychological tests, and implements a post-concussion symptom scale. The newest version of ImPACT is administered through a web browser. It employs keyboard input on a choice reaction time instead of mouse-button input from the desktop version and has shown to yield fewer errors associated with left-right confusion. This ultimately causes fewer invalid results than previous versions [23].

The visual-motor speed (VMS) component of ImPACT is commonly used for determining visual-motor deficits and has been shown to be the most reliable of the ImPACT composite scores. In addition, the reaction time (RT) and visual memory (VIS) composite scores address visual processing and motor speed. These scores provide unique information incorporating visual processing acuity and oculomotor speed. Deficits in VMS, VIS, and RT may reflect axonal damage to oculomotor neurons, but visual processing and performance deficits can have numerous other concussion-related contributing factors [12].

ImPACT is the most widely used computerized neurocognitive assessment in North America [23]. It is internationally used in soccer by the English premiere soccer league as stated in 2017 by Mark Lovell at ImPACT Applications, Inc., San Diego, CA. It has been reported that 93.0% of institutions that use computerized neurocognitive testing use the ImPACT assessment battery. In 2003, approximately 200 high schools, intercollegiate athletic programs, and professional teams were reported to utilize the ImPACT assessment battery [24]. This included nine of 11 Big Ten football teams, the National Football League, Major League Baseball, National Basketball Association, championship auto racing teams, and the US Olympic women’s hockey team [23]. Consequently, by 2017, 7,400 high schools and over 1,000 colleges were reportedly using the ImPACT assessment battery by ImPACT Applications, Inc. Among the institutions that use ImPACT, 27.8% reported that both an athletic trainer and a physician interpret ImPACT results, while 18.1% reported that an athletic trainer alone interprets the test. It was reported that 10.8% of tests were interpreted by a physician alone, and 6.8% of tests were interpreted by a neuropsychologist alone [8]. Athletic trainers who examine the ImPACT data reported a workshop attendance rate of 26.4%, demonstrating its ease of use [8].

The ImPACT screening battery has been found to be sensitive to the acute effects of concussion, revealing substantial changes in functioning in a large percentage of concussed athletes in the first few days post-injury [25]. In particular, Iverson, et al. found that athletes demonstrated a significant decline in verbal memory and visual memory, an increase in symptom reporting, and slower processing speed and reaction times within 72 hours of a concussion [25]. Athletes with concussions were 47 times more likely to have two or more declines across the five composite scores than non-concussed subjects. The authors determined the Pearson test-retest correlation coefficients for the verbal memory, visual memory, reaction time, and processing speed composite scores, which ranged from 0.65 to 0.86. Such test-retest coefficients are comparable to or higher than many other neuropsychological tests [25]. Similarly, Schatz, et al. found that concussed athletes assessed 36 hours, four days, and seven days post-injury performed worse on verbal memory tests, memory and reaction time indices, and reported more symptoms compared to baseline [26].

In a more recent study, one or more false positives on ImPACT testing was found among 38.40% of participants 45 days after injury and among 34.20% of participants 50 days after injury [14]. However, the overall sensitivity and specificity of ImPACT have been determined to be 81.9 - 91.4% and 69.1 - 89.4%, respectively [23, 26]. Interestingly, the ImPACT battery contains criteria that identify invalid performance believed to be due to a variable or insufficient effort on the part of the examinee [27]. Data from ImPACT yielded 94.6% sensitivity and 97.3% specificity among asymptomatic athletes suspected of hiding their concussion. Therefore, Schatz and Sandel concluded that the online version of ImPACT is a valid measure of neurocognitive performance at the acute stages of a concussion. It has high levels of sensitivity and specificity, even when athletes appear to be denying post-concussion symptoms. By attempting to hide the symptoms of a concussion or otherwise look good on the ImPACT test, athletes displayed more variable behavior and paradoxically distinguished themselves from matched controls. This allowed the test to identify their neurocognitive deficits [23]. Those athletes who were more forthcoming with symptom data displayed more normal ranges of behavior, thus overlapping with more normal controls. This resulted in decreased specificity.

The Pediatric ImPACT was developed as a computerized assessment battery for children aged five to 12 years in order to provide developmentally appropriate stimuli and task instructions, factor-derived composite scores, empirically-based clinical algorithms, and comprehensive normative data sets. The six Pediatric ImPACT neurocognitive subtests are based upon the original measure with adaptations of task instructions, cognitive demands, stimuli, and format to make them appropriate for younger children. The reliability and usefulness of the ImPACT test battery as a valid instrument in the evaluation of a sports-related concussion have been confirmed by several sources [16, 23, 25]. In August 2016, the US Food and Drug Administration permitted marketing of the two devices to assess a patient’s cognitive function immediately after a suspected brain injury or concussion: The Immediate Post-Concussion Assessment and Cognitive Testing (ImPACT) and ImPACT Pediatric. They are intended as part of the medical evaluation that doctors perform to assess signs and symptoms of a head injury [28-29].

Concussion Resolution Index (CRI)

HeadMinder, Inc.’s CRI is an online neurocognitive and neurobehavioral assessment tool. The test includes six subtests that evaluate the speed of information processing, visual recognition, and reaction time. Three composite scores are automatically computed: simple reaction time, complex reaction time, and processing speed [30]. Past research has documented the reliability and validity of the CRI. It has been observed to be sensitive in identifying post-concussion symptoms and resistant to retest effects. Specifically, CRI has been described to have an 88% sensitivity to a concussion. However, Broglio, et al. found that on days 45 and 50 after injury, 19.20% and 32.90% of participants had one or more false positives on the CRI, respectively [31].

CogSport

CogState’s CogSport test consists of a series of seven card tasks measuring five composite cognitive domains. These domains are reaction time, decision-making, matching, working memory, and attention. Collie, et al. found that CogSport reliably measures psychomotor function, decision making, working memory, and learning [31-32]. Moreover, CogSport was found to display high correlations with conventional paper and pencil neuropsychological tests of information processing and attention [32]. However, considerable variability in the sensitivity and specificity of the composite scores has been reported [33].

The King-Devick (KD) Oculomotor Test

Neuronal injury resulting from concussive injury can prompt impaired visual movements and oculomotor speed in concussed patients. Poor oculomotor function has been reported as one of the most robust discriminators for the identification of mild traumatic brain injury. It is estimated that oculomotor dysfunction is present to some degree in 65 - 90% of patients who have experienced some form of traumatic brain injury. The visual-motor deficits often reported by such patients include difficulty with saccades, accommodation, smooth pursuit, fixation, reading, and photosensitivity [12]. The KD test is traditionally used to evaluate reading efficiency in children that may be compromised by dyslexia or impaired saccadic eye movements. However, it has recently been promoted as a practical sideline concussion tool for its ease of administration and the rapid manner in which it can be performed. It can usually be given in less than two minutes [12].

Specifically, the KD test requires athletes to read single digit numbers from a series of three cards. The numbers on each card are uniquely arranged and spaced, with a progressive increase in difficulty with each successive card. The athlete holds the cards at a self-chosen comfortable distance and reads the numbers from left to right and top to bottom, as quickly as possible without making an error. The athlete is permitted three attempts to complete each card, and the fastest time without an error is recorded for each card. Each of the best times is summed for a total time.

Tjarks, et al. examined the utility of the KD test by comparing its longitudinal data with PCSS measures and the four composite scores from ImPACT in recently concussed patients. They observed a significant association between KD and ImPACT composite scores, as well as similar tracking in KD performance and PCSS scores [12]. In particular, KD times and PCSS scores progressively decreased over the course of four visits, and three of the ImPACT composite scores increased over the four visits. Reaction time progressively decreased. These results are consistent with the notion that participants were progressively recovering from their brain injuries across the period of the study. Moreover, they support the clinical utility of the KD test in acute concussion diagnosis [12].

In a prospective observational cohort study, 22 concussion events were recorded. Notably, only five concussive incidents were witnessed, and the remaining 17 unrecognized concussive incidents were identified with KD testing [34]. KD was able to identify players that had not shown or reported any signs or symptoms of concussion, but who had a meaningful head injury. Thus, the authors concluded that KD is suitable for rapid assessment in a limited time frame on the sideline to assess and review suspected concussed players. The individuals with unrecognized concussions identified with KD on average presented fewer symptoms, lower symptom severity, better balance examination, and better immediate and delayed memory scores than those with witnessed concussions. However, none of these differences were significant.

Advantages of the KD test include its relatively low cost and a minimal level of expertise required to administer the test. KD tests for impairment of eye movement, attention, language, and other areas that correlate with suboptimal brain function that may occur following a concussive episode [34]. Given that ImPACT, SCAT2, SAC, and CogSport do not assess eye movements or brainstem function well, the KD test may serve as an effective clinical tool to assess athletes with suspected concussion.

The advantages and disadvantages of each test are summarized in Table 1.

**Table 1:** Comparison of Concussion Assessment Tools PCSS: Post-Concussion Symptom Scale; SAC: Standard Assessment of Concussion; SCAT3: Standard Concussion Assessment Tool; ImPACT: Immediate Post-Concussion Assessment and Cognitive Testing; CRI: Concussion Resolution Index; KD: King-Devick

Test	Advantages	Disadvantages
PCSS	Large battery of concussion-related symptoms tests	Subjective self-reported questionnaire; possible wide variability in results
SAC	Ease of administration (paper and pencil); high sensitivity and specificity	Cannot be used for continued monitoring due to rapid return to baseline (usually within 48 hours post-concussion)
SCAT3	Wide variety of symptoms tested (including all symptoms in PCSS); separate version for children	Not a comprehensive neuropsychological test and therefore cannot be used alone
ImPACT	Comprehensive test with high sensitivity and specificity; can be used as a standalone test; can identify athletes attempting to hide symptoms; can be used for -longer-term monitoring; separate version for children	Athletes more forthcoming with symptoms may display more normal behavior and decrease sensitivity of test
CRI	Highly sensitive and resistant to retest effects	Cannot be used for longer-term monitoring (many false positives on later tests)
CogSport	High correlations with paper and pencil neuropsychological tests	Reportedly high variability in sensitivity and specificity
KD	Easy to administer; tests eye movement and brainstem functions that other tests do not; able to identify events in athletes without symptoms of concussion (unrecognized concussions)	Not a comprehensive neuropsychological test; does not test many of the classic concussion symptoms

## Conclusions

Many diagnostic modalities can be utilized for the diagnosis and evaluation of concussions. However, no single test has proven sufficient for stand-alone use in the diagnosis of sports-related concussions. Because of the limitations of available concussion rating scales, there is an important need to assess other objective methods for concussion diagnosis and evaluation. Methods are needed that will help stratify the injuries both for the individual event as well as longitudinally over the patient’s lifetime. Due to the individualized nature of each concussion, post-injury assessment and management require a complex understanding of both clinical and non-clinical factors. Though the available assessment tools may facilitate the evaluation of concussive injury and subsequent return-to-play decisions, the psychometrics, test setting, administrators, and other individual characteristics of the athletes contribute a substantial number of issues that must be taken into consideration. Healthcare providers involved in the evaluation of sports-related concussion should understand the influence of such factors and manage decisions accordingly. Given that concussive injury is dynamic and highly personalized, skill, experience, and flexibility on the part of the clinician are essential in guiding effective management of injured athletes.
